# Sarcopenia and sarcopenic obesity are independent adverse prognostic factors in resectable pancreatic ductal adenocarcinoma

**DOI:** 10.1371/journal.pone.0215915

**Published:** 2019-05-06

**Authors:** Elisabeth S. Gruber, Gerd Jomrich, Dietmar Tamandl, Michael Gnant, Martin Schindl, Klaus Sahora

**Affiliations:** 1 Division of General Surgery, Department of Surgery, Pancreatic Cancer Unit, Comprehensive Cancer Center, Medical University of Vienna, Vienna, Austria; 2 Department of Biomedical Imaging and Image-guided Therapy, Medical University of Vienna, Vienna, Austria; Centro Nacional de Investigaciones Oncologicas, SPAIN

## Abstract

**Background:**

Incidence and mortality of pancreatic ductal adenocarcinoma (PDAC) are on the rise. Sarcopenia and sarcopenic obesity have proven to be prognostic factors in different types of cancers. In the context of previous findings, we evaluated the impact of body composition in patients undergoing surgery in a national pancreatic center.

**Methods:**

Patient’s body composition (n = 133) was analyzed on diagnostic CT scans and defined as follows: Skeletal muscle index ≤38.5 cm^2^/m^2^ (women), ≤52.4 cm^2^/m^2^ (men); obesity was classified as BMI ≥25kg/m^2^.

**Results:**

Sarcopenia showed a negative impact on overall survival (OS; 14 vs. 20 months, *p =* 0.016). Sarcopenic patients suffering from obesity showed poorer OS compared to non-sarcopenic obese patients (14 vs. 23 months, *p =* 0.007). Both sarcopenia and sarcopenic obesity were associated with sex (*p<*0.001 and *p =* 0.006; males vs. females 20% vs. 38% and 12% vs. 38%, respectively); sarcopenia was further associated with neoadjuvant treatment (*p =* 0.025), tumor grade (*p =* 0.023), weight loss (*p =* 0.02) and nutritional depletion (albumin, *p =* 0.011) as well as low BMI (*<*25 kg/m^2^, *p =* 0.038). Sarcopenic obese patients showed higher incidence of major postoperative complications (*p<*0.001). In addition, sarcopenia proved as an independent prognostic factor for OS (*p =* 0.031) in the multivariable Cox Regression model.

**Conclusion:**

Patients with sarcopenia and sarcopenic obesity undergoing resection for PDAC have a significantly shorter overall survival and a higher complication rate. The assessment of body composition in these patients may provide a broader understanding of patients’ individual condition and guide specific supportive strategies in patients at risk.

## Introduction

Pancreatic cancer is one of the most lethal malignancies. Even in times of multimodal treatment the prognosis remains poor[1]. In search of prognostic factors, the main focus lies on tumor-specific factors (TNM/UICC) rather than taking host-specific conditions into account[2]. Recently, body composition was evaluated in different oncologic patient cohorts; hereby, sarcopenia proved as a prognostic factor of morbidity, mortality and survival, especially in combination with obesity. It has also been associated with impaired response to chemo- and radiotherapy in a variety of cancers[3–6]. These insights started intensive research on therapeutic strategies including nutritional and pharmacological support as well as physical exercise[7–10]. Guidelines were elaborated to assess sex-specific body composition by measuring the cross-sectional muscle and visceral fat area at the level of the third lumbar vertebra on routinely available diagnostic computed tomography images and to analyze data using validated software[11–15]. Yet, in patients with pancreatic ductal adenocarcinoma (PDAC), data was controversially discussed[16–26]. However, a recent meta-analysis showed that sarcopenia and sarcopenic obesity was significantly associated with poorer overall survival; in patients with resectable PDAC, data on the impact of body composition on treatment-relevant postoperative complications are still rare, thus no conclusion could be drawn from these studies[27].

In the context of these previous findings, we aimed to explore the prevalence and clinical implications of sarcopenia and sarcopenic obesity in a patient cohort that underwent pancreatic resection in a national leading centre for PDAC treatment. Here we investigated the impact on survival as well as the relationship to nutritional status, conventional patient and tumor characteristics, comorbidities and postoperative complications.

## Materials and methods

### Ethical approval

We performed a retrospective analysis by summarizing patient and tumor characteristics anonymously from our institutional database at the Medical University of Vienna. Approval was obtained from the local Institutional Review Board (”Ethik Kommission”), Medical University of Vienna (IRB protocol #2012-P-000619/1).

### Data collection

Patients who underwent pancreatic resection for PDAC between 2005 and 2010 were identified. Patients who received neoadjuvant chemo- or radiochemotherapy of various protocols due to borderline resectable disease were also included. After completion of neoadjuvant treatment, restaging scans were reviewed by an interdisciplinary panel/tumorboard. If resectable, patients were allocated to either pylorus-preserving pancreaticoduodenectomy or distal pancreatectomy depending on the location of the tumor. Patients with reported stable or progressive disease, despite completed neoadjuvant treatment who were directed towards further oncological treatment, were excluded from the study. Of these patients, those with preoperative diagnostic contrast enhanced computed tomographic (CT) scans, taken within 6 weeks prior to surgery, available in the electronic radiologic database, were included. Eligible patients were followed-up on for at least 24 months after surgery. Follow-up was conducted mostly within our outpatient clinic; for those who were followed-up on elsewhere, information was assessed via the Statistic Austria Death Index. If not available, patients or relatives were contacted via telephone and personally surveyed about the follow-up status.

Patient and tumor characteristics were recorded retrospectively. Variables included sex, age at the time of surgery, personal medical history, physical examination as well as routine laboratory testing. In addition, details on the surgical procedure, the postoperative course and the final pathological report were conducted. All data were extracted from our institutional database. Comorbidities were assessed using the Charlson age comorbidity index (CACI)[28], perioperative complications were reported according to the classification system defined by Clavien et al.[29] and fistulas were graded according to the latest consensus definitions[30]. Depleted nutritional status was defined as albumin *<*35mg/dl[31] and weight loss as >5% within 6 months prior to surgery[12]. Only patients whose data were completely available were selected for inclusion.

### Anthropometric measures

Determination of body composition was conducted on preoperative contrast-enhanced, portal-venous-phase CT images as described by Mourtzakis et al.[11]. Images were analyzed using Osirix V5.8 software (Pixmeo Sarl, Switzerland).

**Fig 1:**
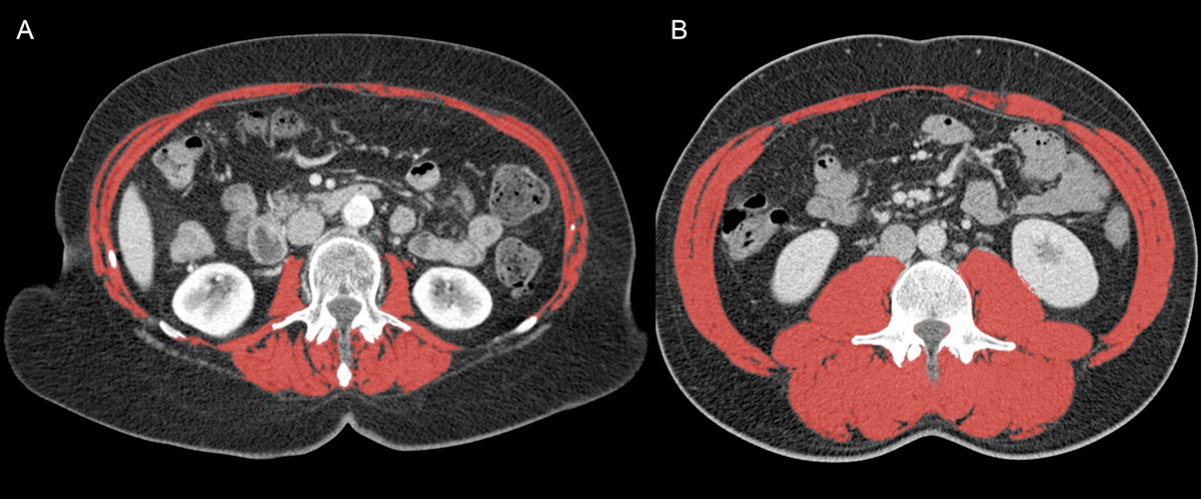
Determination of body composition in patients with resectable pancreatic ductal adenocarcinoma. Axial contrast-enhanced portal-venous phase CT image at the L3 level of a sarcopenic obese patient (A, 67-year old female, BMI 33 kg/m^2^, total muscle tissue cross-sectional area 109.2 cm^2^, skeletal muscle index 39.3 cm^2^/m^2^) compared to a non-sarcopenic obese patient (B, 71-year old female, BMI 31 kg/m^2^, total muscle tissue cross-sectional area 130.4 cm^2^, skeletal muscle index 47.7 cm^2^/m^2^). Marked red: psoas, paraspinal, transverse abdominal, external oblique, internal oblique and rectus abdominis muscles.

CT Hounsfield unit thresholds were -29 to +150 for lean muscle (**Fig 1**)[32] and -190 to -30 HU and -150 to -50 HU for subcutaneous and intramuscular fat and visceral obese tissue[33], respectively. As necessary, a single-trained observer manually corrected the tissue boundaries.

Two consecutive transverse CT images extending from the third lumbar vertebrae (L3) in the inferior direction were analyzed for total lean tissue, total lean muscle (psoas, erector spinae, quadratus lumborum, transverse abdominis, external and internal obliques and rectus abdominis) and adipose tissue (subcutaneous, intramuscular and visceral). Total fat-free tissue comprised soft tissue excluding intestinal contents and bones. In addition, tissue cross-sectional areas (cm^2^) were computed automatically by summing tissue pixels and multiplying by pixel surface area. Areas were normalized for stature (cm^2^/m^2^). Cut-offs for sarcopenia were based on the computed tomography-based sarcopenic obesity study of cancer patients conducted by Prado et al. (i.e., L3 skeletal muscle index ≤38.5 cm^2^/m^2^ for women and ≤52.4 cm^2^/m^2^ for men)[34]. Assessment of total body fat-free mass and total body fat mass was performed using the following regression equations defined by Mourtzakis et al.[11] Total body fat-free mass (FFM) (kg) = 0.3 × [skeletal muscle at L3 (cm^2^)] + 6.06 (r = 0.94); total body fat mass (FM) (kg) = 0.042 × [total adipose tissue at L3 (cm^2^)] + 11.2 (r = 0.88). Cut-off points for overweight and obesity were used as proposed by the World Health Organization (overweight—BMI ≥25kg/m^2^, obese—BMI ≥30kg/m^2^)[35].

### Statistical analysis

Categorical variables were described by frequencies, numerical variables by median (range). For comparison of categorical variables between patient groups a Chi-squared test was used. Numerical variables were compared using the Mann-Whitney U test or a two-sample Student’s t-test, if normally distributed. Overall survival was defined as time of initial PDAC diagnosis until death from PDAC and recurrence-free survival (RFS) was defined as time from PDAC surgery until diagnosis of PDAC recurrence. Survival estimates were calculated by the Kaplan-Meier method (log-rank test for group comparisons). To estimate the median follow-up time, the reversed Kaplan-Meier method was used. Cox regression analysis was performed to estimate group differences between relative risks of death (hazard ratios, 95% confidence interval) and to adjust for effects. Established prognostic factors known to influence PDAC survival (T stage, N stage, grading) were included into the model next to sarcopenia. For explicit examination of the influence of sex and BMI on sarcopenia’s strength for survival prognostication, an interaction model was implemented. A two-sided p-value less than 0.05 was considered statistically significant. The SPSS version 24 software for Mac OsX and Sierra (IBM, USA) was used for analyses.

## Results

### Patient and tumor characteristics

A total of 179 patients underwent surgery for PDAC during the study period. In 46 patients, preoperative CT scans were not available due to technical reasons and were thus excluded from the study. Of the remaining 133 patients, analyzed data were fully available and they were thus included in the study. Of these, 78 patients suffered from sarcopenia and 25 from sarcopenic obesity. Sarcopenia was associated with sex (*p<*0.001), weight loss (*p =* 0.02), lower albumin levels (*p =* 0.011), higher tumor grading (*p =* 0.023) and neoadjuvant therapy (*p =* 0.035). Sarcopenic obesity was associated with sex (*p =* 0.006) and major postoperative complications (*p<*0.001). Patient and tumor characteristics are compiled in **Tables 1 and 2**.

**Table 1:** Patient and tumor characteristics comparing sarcopenic and non-sarcopenic patients.

	Total	Sarcopenia	No Sarcopenia	*p*-value
	n = 133 (100)	n = 78 (58.6)	n = 55 (41.4)	
Age	65 (34–87)	66 (45–84)	65 (34–87)	n.s.
Female	65 (48.9)	27 (20.3)	38 (28.6)	*<*0.001
Male	68 (51.1)	51 (38.3)	17 (12.8)	
CACI* ≥5	20 (15.2)	12 (9.1)	8 (6.1)	n.s.
Postoperative complications (Clavien-Dindo ≥IIIB)	20 (15.0)	13 (9.8)	7 (5.3)	n.s.
Fistula (≥grade B)	27 (20.3)	15 (11.3)	12 (9.0)	n.s.
Weight loss (>5%)	72 (54.1)	49 (36.8)	23 (17.3)	0.017
Albumin (g/l)	40 (19–49)	39 (24–48)	41 (19–49)	0.011
CA 19–9 (kU/l)	140 (0–2953)	174 (0–2953)	125 (0.7–2953)	n.s.
PPPD**	112 (84.2)	68 (51.1)	44 (33.1)	n.s.
Distal PD***	21 (15.8)	10 (7.5)	11 (8.3)	
Neoadjuvant therapy	20 (15.2)	16 (12.1)	4 (3.0)	0.035
Tumor grading (G3)	54 (40.6)	38 (28.6)	16 (12.0)	0.023
Tumor stage ≥T3	108 (81.2)	64 (48.1)	44 (33.1)	n.s.
Nodal metastases (N1)	91 (68.4)	54 (40.6)	37 (27.8)	n.s.
Distant metastases (M1)	7 (5.3)	4 (3.0)	3 (2.3)	n.s.
UICC ≥IIB	94 (70.7)	55 (41.4)	39 (29.3)	n.s.
Resection margin (R1)	22 (16.5)	12 (9.0)	10 (7.5)	n.s.

*Charlson Age-Comorbidity Index

**pylorus-preserving pancreaticoduodenectomy

***pancreatectomy; n.s. denotes not significant; nominal/ordinal variables are expressed by numbers and percentages, continuous variables by median and range.

**Table 2:** Patient and tumor characteristics comparing sarcopenic obese and non-sarcopenic obese patients.

	Total	Sarcopenic obesity	No sarcopenic obesity	*p*-value
	n = 133 (100)	n = 34 (25.6)	n = 99 (74.4)	
Age	68 (34–87)	25 (45–84)	108 (34–87)	n.s.
Female	65 (48.9)	8 (12.3)	57 (87.7)	0.006
Male	68 (51.1)	26 (38.2)	42 (61.8)	
CACI* ≥5	20 (15.2)	1 (0.8)	19 (14.4)	n.s.
Postoperative complications (Clavien-Dindo ≥IIIB)	20 (15.0)	18 (13.5)	2 (1.5)	*<*0.001
Fistula (≥grade B)	27 (20.3)	4 (3.0)	23 171.3)	n.s.
Weight loss (>5%)	72 (54.1)	16 (12.0)	56 (42.1)	n.s.
Albumin (g/l)	40 (19–49)	39 (24–48)	35 (19–49)	n.s.
CA 19–9 (kU/l)	81 (0–2953)	22 (0–2953)	59 (0.7–2953)	n.s.
PPPD**	112 (84.2)	22 (16.5)	90 (67.7)	n.s.
Distal PD***	21 (15.8)	3 (2.3)	18 (13.5)	
Neoadjuvant therapy	20 (15.2)	5 (3.8)	15 (11.4)	n.s.
Tumor grading G3	54 (40.6)	13 (9.8)	14 (30.8)	n.s.
Tumor stage ≥T3	108 (81.2)	20 (15.0)	88 (66.2)	n.s.
Nodal metastases (N1)	91 (68.4)	17 (12.8)	74 (55.6)	n.s.
Distant metastases (M1)	7 (5.3)	2 (1.5)	5 (3.8)	n.s.
UICC ≥IIB	94 (70.6)	18 (13.5)	76 (57.1)	n.s.
Resection margin (R1)	22 (16.5)	6 (4.5)	16 (12.0)	n.s.

*Charlson Age-Comorbidity Index

**pylorus-preserving pancreaticoduodenectomy

***pancreatectomy; n.s. denotes not significant; nominal/ordinal variables are expressed by numbers and percentages, continuous variables by median and range.

### Perioperative features

The majority of patients (112, 84.2%) underwent pylorus-preserving pancreaticoduodenectomy, (21, 15.8%) received distal pancreatectomy. Tangential or segmental venous resections were necessary in 22 (16%) patients. Operative mortality was 4%. The median length of stay was 13 days (6–85 days). There was no correlation between sarcopenia or sarcopenic obesity and postoperative morbidity (sarcopenia vs. no sarcopenia: 9.8% vs. 5.3%, *p =* 0.531 and sarcopenic obesity vs. no sarcopenic obesity: 7.5% vs. 9.5%, *p =* 0.876), mortality (sarcopenia vs. no sarcopenia: 3% vs. 0.8%, *p =* 0.323 and sarcopenic obesity vs. no sarcopenic obesity: 1.5% vs. 2.3%, *p =* 0.216) or median length of stay (sarcopenia vs. no sarcopenia: 14 vs. 11 days, *p =* 0.243 and sarcopenic obesity vs. no sarcopenic obesity: 16 vs. 13 days, *p =* 0.435).

### Body composition analysis

Data on sarcopenia and sarcopenic obesity in relation to patient and tumor characteristics are shown in **Tables 1 and 2**. Sarcopenic obesity is defined as the presence of sarcopenia in patients with a BMI ≥30 kg/m^2^ according to Prado et al.[34]. Due to the relatively small number of obese patients (n = 7, 5%) in our cohort, we decided to fuse the WHO-defined groups “overweight” (BMI ≥25 kg/m^2^) and “obese” (BMI ≥30 kg/m2)[35]. Data on body composition in relation to sarcopenia are shown in **Table 3**. Due to significant sex differences between non-sarcopenic and sarcopenic as well as sarcopenic obese patients (*p<*0.001, **Table 1 and** *p =* 0.006, **Table 2**, respectively), body composition data are described separately in **Table 4**.

**Table 3:** Body composition data comparing sarcopenic and non-sarcopenic patients.

	Total	Sarcopenia	No Sarcopenia	*p*-value
	n = 133 (100)	n = 78 (58.6)	n = 55 (41.4)	
**WHO classification, n (%)**				
Mean BMI (kg/m^2^)	24 (±4.3)	24 (±4.2)	25 (±4.4)	0.058
Normal/underweight (BMI *<*25 kg/m^2^)	65 (48.9)	44 (33.1)	21 (15.8)	0.038
Overweight/obese (BMI ≥25 kg/m2)	68 (51.2)	34 (25.6)	34 (25.6)	
**Absolute body composition data, cm**^**2**^ **(**±**SD)**				
Lumbar total muscle tissue cross-sectional area	126.5 (±28.6)	122 (±27.9)	132 (±28.7)	n.s.
Lumbar total adipose tissue cross-sectional area	141.7 (±99.7)	145.2 (±101.8)	136.7 (±97.4)	n.s.
**Relative body composition data, related to body surface area, cm**^**2**^**/m**^**2**^ **(**±**SD)**				
Lumbar skeletal muscle index	43.8 (±7.8)	40.7 (±6.7)	48.3 (±7.2)	n.s.
Lumbar adipose tissue index	49.2 (±34.4)	48.2 (±31.9)	50.7 (±37.9)	n.s.
**Estimated body composition data, kg (**±**SD)**				
Estimated total fat-free mass	44 (±8.6)	42.7 (±8.4)	45.9 (±8.6)	n.s.
Estimated total fat mass	17.1 (±4.2)	17.3 (±4.3)	16.9 (±4.1)	n.s.

n.s. denotes not significant; ±SD denotes standard deviation.

**Table 4:** Overall and sex-specific body composition data.

Body composition	Cohort	Overall	Sarcopenia	No Sarcopenia
	Overall	n = 133 (100)	n = 78 (58.6)	n = 55 (41.4)
	Female	n = 65 (48.9)	n = 27 (20.3)	n = 38 (28.6)
	Male	n = 68 (51.1)	n = 51 (38.3)	n = 17 (12.8)
**WHO classification, n (%)**				
Mean BMI (kg/m^2^)	Overall	24 (±4.3)	24 (±4.2)	25 (±4.4)
	Female	24 (±4.7)	22.7 (±4.1)	24.8 (±4.9)
	Male	25 (±3.9)	24.5 (±4.1)	26.1 (±2.8)
Normal/underweight (BMI <25 kg/m2)	Overall	65 (48.9)	44 (33.1)	21 (15.8)
	Female	37 (56.9)	19 (29.2)	18 (27.2)
	Male	28 (41.2)	25 (36.8)	3 (4.4)
Overweight/obese (BMI ≥25 kg/m2)	Overall	68 (51.2)	34 (25.6)	34 (25.6)
	Female	28 (43.1)	8 (12.3)	20 (30.8)
	Male	40 (58.8)	26 (38.2)	14 (20.6)
**Absolute body composition data, cm**^**2**^ **(**±**SD)**				
Lumbar total muscle tissue cross-sectional area	Overall	126.5 (±28.6)	122 (±27.9)	132 (±28.7)
	Female	106.2 (±17.6)	91.9 (±12.1)	116.3 (±13.3)
	Male	146.0 (±23.1)	138.1 (±19.3)	169.5 (±17.2)
Lumbar total adipose tissue cross-sectional area	Overall	141.7 (±99.7)	145.2 (±101.8)	136.7 (±97.4)
	Female	110.0 (±83.4)	195.3 (±65.4)	120.4 (±93.6)
	Male	172.1 (±105.1)	171.7 (±108.1)	173.4 (±98.5)
**Relative body composition data, related to body surface area, cm**^**2**^**/m**^**2**^ **(**±**SD)**				
Lumbar skeletal muscle index	Overall	43.8 (±7.8)	40.7 (±6.7)	48.3 (±7.2)
	Female	40.1 (±6.8)	34.0 (±4.0)	44.5 (±4.6)
	Male	47.5 (±7.0)	44.4 (±4.7)	56.9 (±3.2)
Lumbar adipose tissue index	Overall	49.2 (±34.4)	48.2 (±31.9)	50.7 (±37.9)
	Female	44.3 (±13.9)	25.3 (±24.5)	46.9 (±38.7)
	Male	44.1 (±14.0)	43.4 (±15.4)	46.0 (±8.6)
**Estimated body composition data, kg (**±**SD)**				
Estimated total fat-free mass	Overall	44 (±8.6)	42.7 (±8.4)	45.9 (±8.6)
	Female	37.9 (±5.3)	33.6 (±3.6)	40.9 (±4.0)
	Male	50.0 (±6.9)	47.5 (±5.8)	56.9 (±5.2)
Estimated total fat mass	Overall	17.1 (±4.2)	17.3 (±4.3)	16.9 (±4.1)
	Female	15.8 (±3.5)	15.2 (±2.8)	16.3 (±3.9)
	Male	18.4 (±4.4)	8.4 (±4.5)	18.5 (±0.6)

n.s. denotes not significant; ±SD denotes standard deviation.

### Sarcopenia, sarcopenic obesity and survival

**Fig 2:**
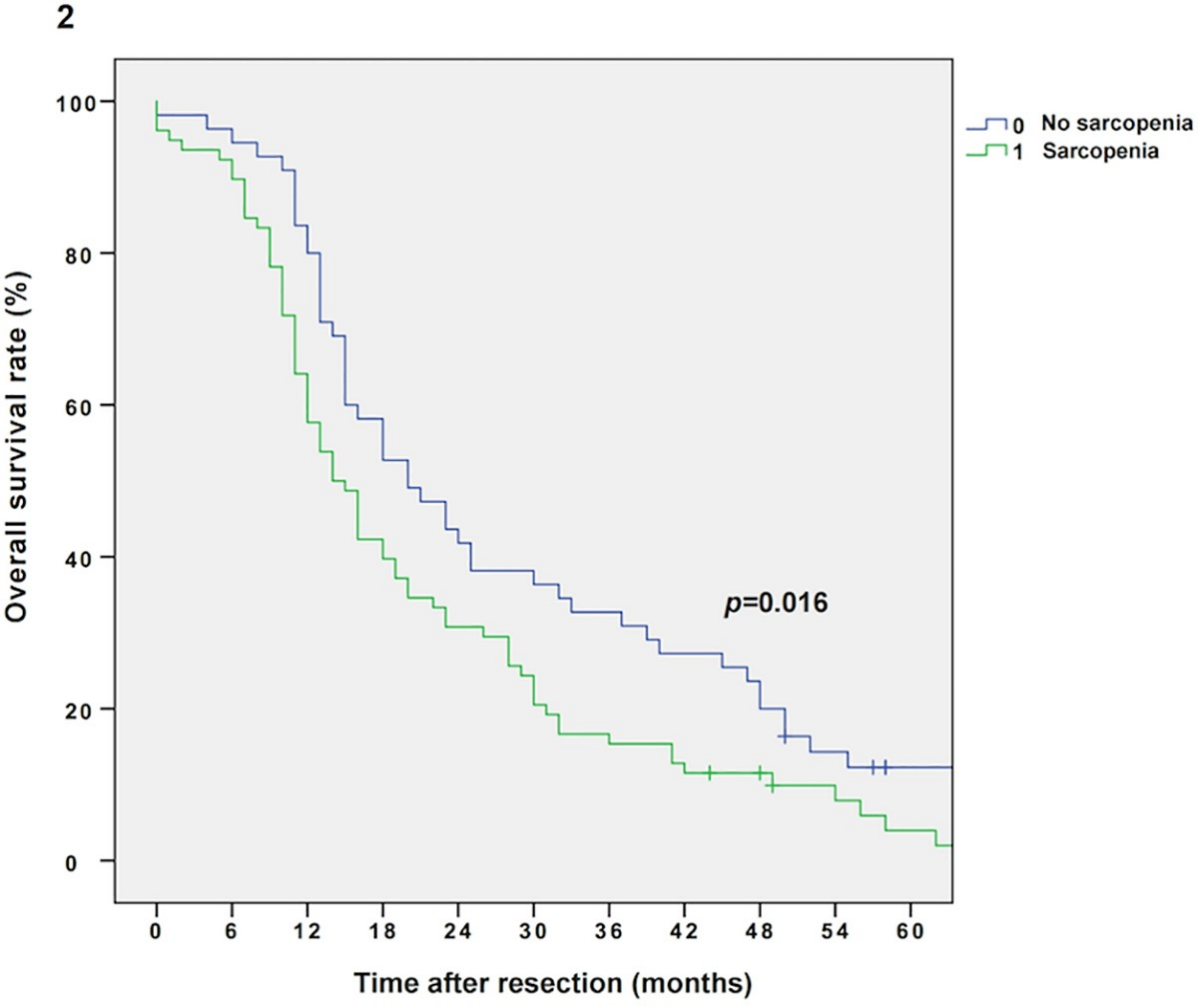
Kaplan Meier curve for overall survival in sarcopenic vs. non-sarcopenic patients. Sarcopenia diminishes overall survival in patients with resectable PDAC (14 vs. 20 months, *p =* 0.016).

**Fig 3:**
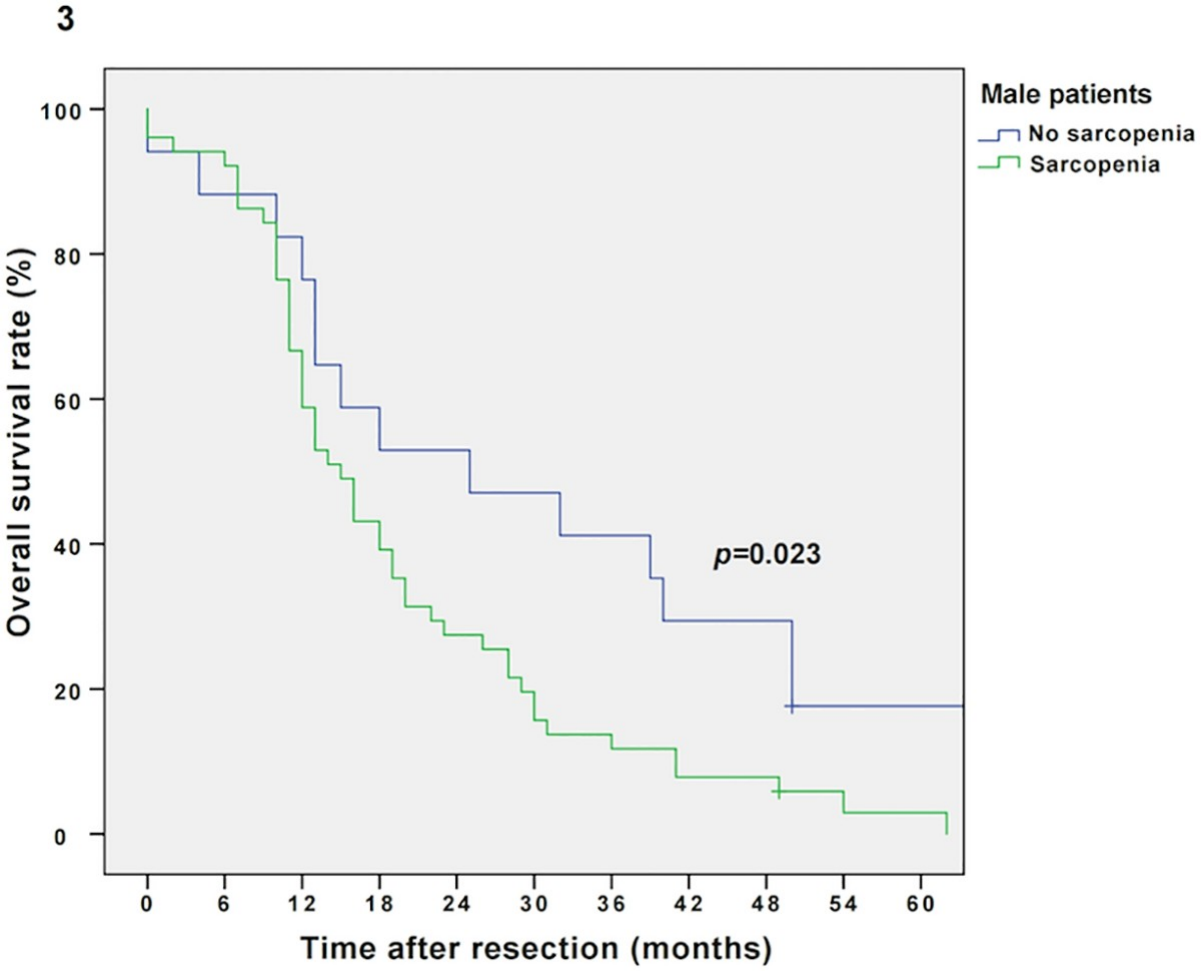
Kaplan Meier curve for overall survival in sarcopenic vs. non-sarcopenic male patients. Sarcopenia diminishes overall survival in male patients with resectable PDAC (15 vs. 25 months, *p =* 0.023).

**Fig 4:**
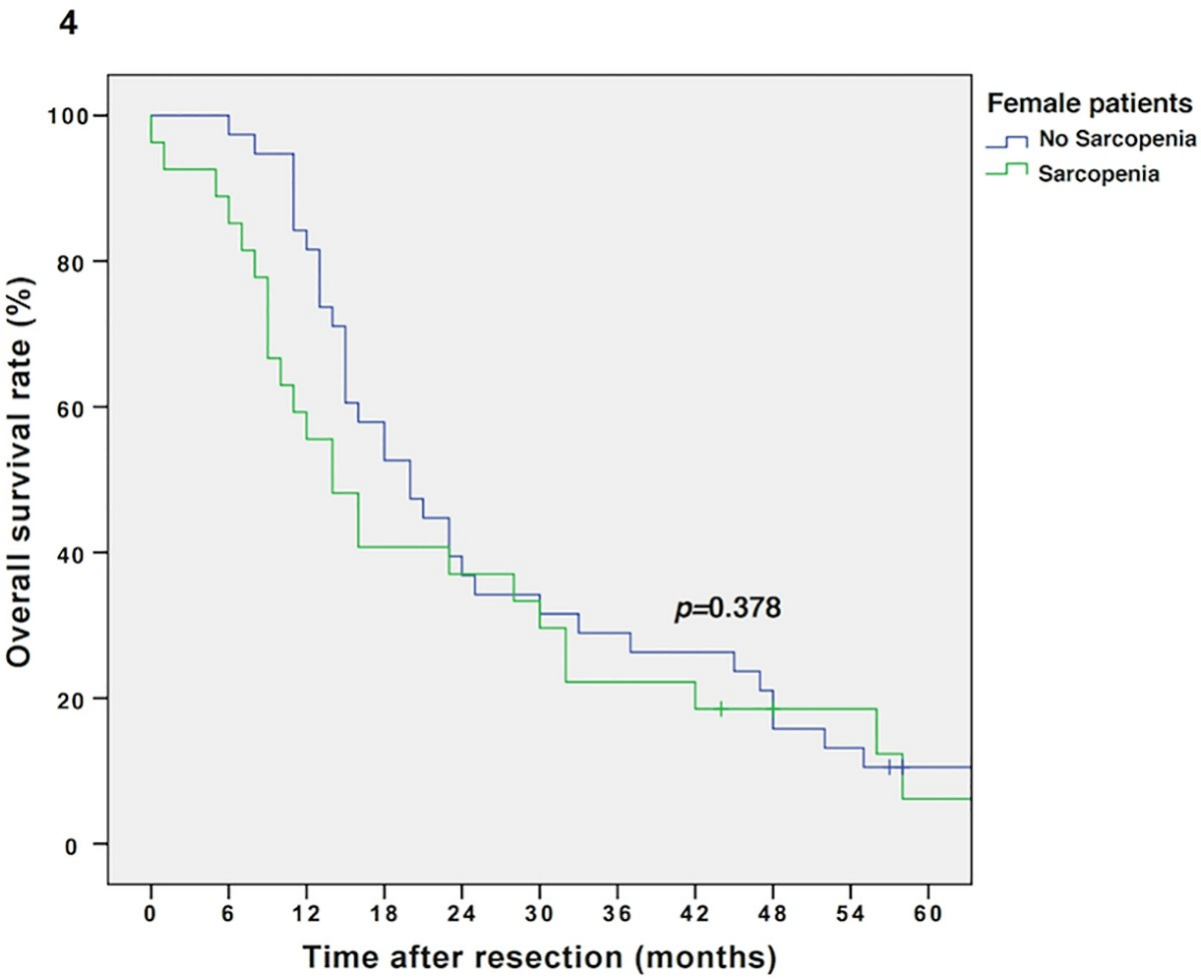
Kaplan Meier curve for overall survival in sarcopenic vs. non-sarcopenic female patients. Sarcopenia does not diminish overall survival in female patients with resectable PDAC (14 vs. 20 months, *p =* 0.378).

**Fig 5:**
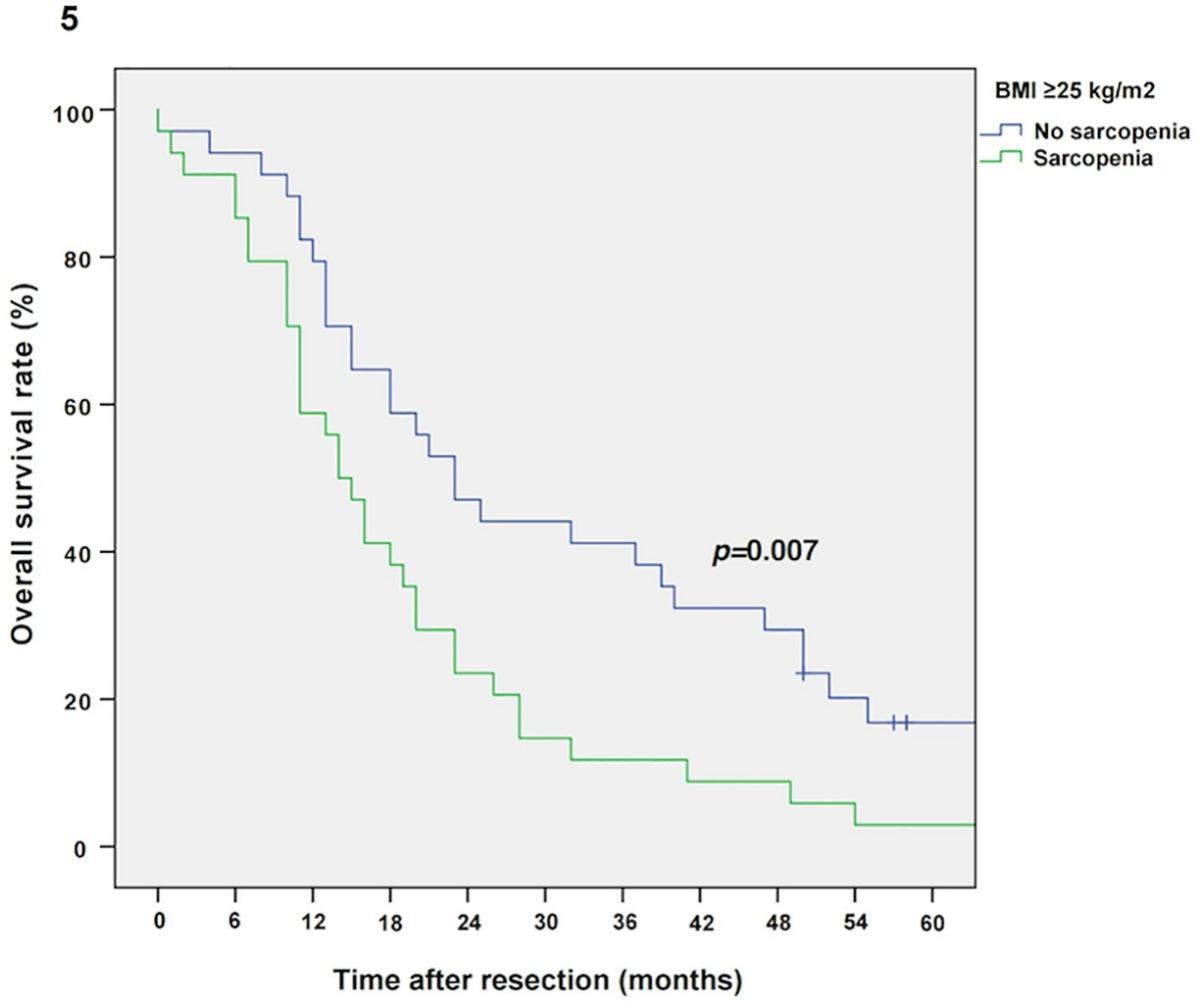
Kaplan Meier curve for overall survival in obese sarcopenic vs. non-sarcopenic patients (BMI ≥25 kg/m^2^). Sarcopenia diminishes overall survival in obese patients with resectable PDAC (14 vs. 23 months, *p =* 0.007).

**Fig 6:**
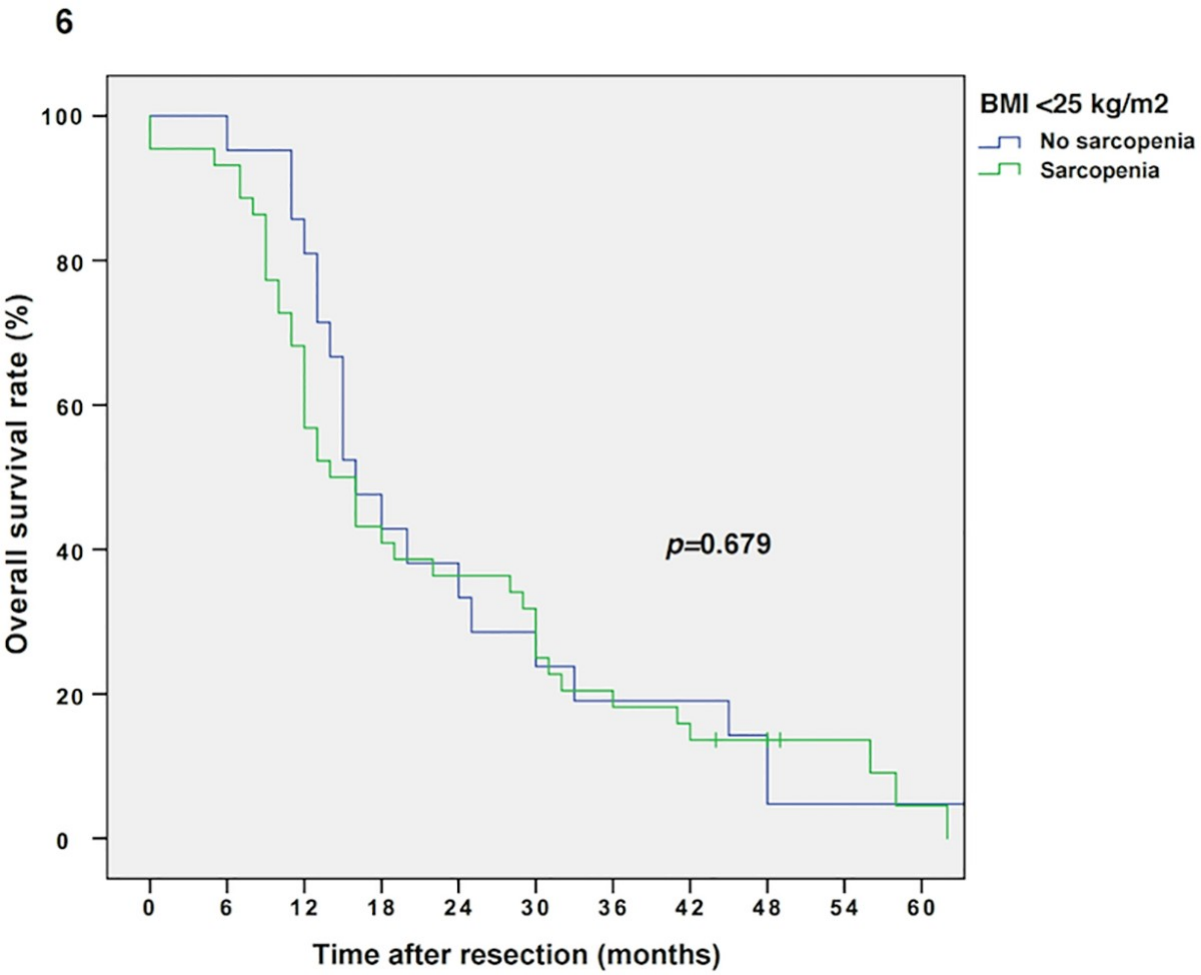
Kaplan Meier curve for overall survival in normal/underweight sarcopenic vs. non-sarcopenic patients (BMI *<*25 kg/m^2^). Sarcopenia does not diminish overall survival in normal/underweight patients with resectable PDAC (14 vs. 16 months, *p =* 0.679).

The median follow-up was 134 months, the median survival was 16 months (13–19 months) in the entire cohort and 103 patients (77%) relapsed during the follow-up period. Sarcopenic patients showed impaired overall survival (OS) compared to non-sarcopenic patients (Kaplan Meier/log rank; 14 vs. 20 months, *p =* 0.016, **Fig 2).** Multivariable Cox regression analysis revealed sarcopenia as an independent prognostic factor for OS (*p =* 0.031, adjusted for tumor grading, stage and nodal metastases; *p =* 0.023, adjusted for tumor grading/stage and nodal metastases as well as BMI; *p =* 0.045, adjusted for tumor grading/stage and nodal metastases as well as sex). Furthermore, as demonstrated by an interaction model, the prognostic effect of sarcopenia is modified by the patient’s BMI level and sex (*p*-value for interaction term: *p =* 0.018 and *p =* 0.032, respectively). Thus, survival analysis for sarcopenic vs. non-sarcopenic patients was performed separately for sexes (Kaplan Meier/log rank: male sarcopenic vs. non-sarcopenic: 15 vs. 25 months, *p =* 0.023, and female sarcopenic vs. non-sarcopenic: 14 vs. 20 months, *p =* 0.387, respectively; **Figs 3 and 4**) and BMI groups (Kaplan Meier/log rank: BMI ≥25 kg/m^2^ (sarcopenic obese vs. non-sarcopenic obese): 14 vs. 23 months, *p =* 0.007 and BMI *<*25 kg/m^2^ (sarcopenic normal/underweight vs. non-sarcopenic normal/underweight): 14 vs. 16 months, *p =* 0.679, respectively; **Figs 5 and 6**). Overall survival data, according to Kaplan Meier and Cox Regression analysis are compiled in **Tables 5 and 6**.

**Table 5:** Kaplan Meier estimates for overall survival.

Kaplan Meier estimates	Median (range)	*p*-value
**Total (n = 133)**	16 months (13–19)	n.a.
**Sarcopenia**		
Yes (n = 78) vs. no (n = 55)	14 (11–17) vs. 20 (14–26)	0.016
**Sex-specific sarcopenia**		
Female: Yes (n = 27) vs. no (n = 38)	14 (9–19) vs. 20 (14–26)	n.s.
Male: Yes (n = 51) vs. no (n = 17)	15 (12–19) vs. 25 (2–48)	0.023
**Sarcopenic obesity** (BMI ≥25 kg/m^2^)		
Yes (n = 25) vs. no (n = 108)	14 (11–17) vs. 23 (16–30)	0.007
**Serum albumin**		
*<*35 (n = 18) vs. ≥35 mg/dl (n = 115)	12 (9–15) vs. 18 (14–21)	0.044
**Neoadjuvant therapy**		
Yes (n = 113) vs. no (n = 20)	28 (22–25) vs. 24 (16–32)	n.s.
CACI*		
≥5 (n = 43) vs. *<*5 (n = 90)	15 (12–18) vs. 18 (14–22)	0.032
**Tumor grading**		
G3 (n = 54) vs. G1-2 (n = 79)	20 (16–24) vs. 12 (11–13)	0.012
**Tumor stage**		
T≥3 (n = 108) vs. T*<*3 (n = 25)	23 (13–33) vs. 16 (13–19)	n.s.
**Nodal metastases**		
N1 (n = 91) vs. N0 (n = 42)	20 (8–32) vs. 16 (13–19)	0.005
**Distant metastases**		
M1 (n = 7) vs. M0 (n = 126)	16 (13–19) vs. 13 (5–21)	n.s.
**Resection margin**		
R1 (n = 22) vs. R0 (n = 111)	18 (14–21) vs. 15 (12–18)	n.s.

*Charlson Age-Comorbidity Index; n.a. denotes not applicable; n.s. denotes not significant.

**Table 6:** Uni- and multivariable Cox-Regression analyses and interaction models for overall survival.

**Univariable analysis**	**HR (95% CI)**	***p*-value**
Sarcopenia	1.55 (1.07–2.23)	0.016
Sarcopenic obesity	1.02 (1.00–1.03)	0.007
Serum albumin	1.63 (1.0–2.7)	0.044
BMI	0.86 (0.60–1.22)	n.s.
CACI*	1.68 (1.03–2.73)	0.032
Neoadjuvant therapy	1.0 (0.6–1.62)	n.s.
Tumor grading	1.16 (1.03–1.31)	0.012
Tumor stage	1.41 (1.0–2.0)	n.s.
Nodal metastases	1.75 (1.17–2.62)	0.005
Distant metastases	1.04 (0.46–2.36)	n.s.
Resection margin	1.47 (0.91–2.35)	n.s.
**Multivariable models**	**HR (95% CI)**	***p*-value**
Sarcopenia**	1.51 (1.04–2.19)	0.031
Tumor grading	1.17 (1.04–1.33)	0.011
Tumor stage	1.3 (0.92–1.85)	n.s.
Nodal metastases	1.76 (1.16–2.66)	0.008
Sarcopenic obesity**	1.46 (0.92–2.3)	n.s.
Tumor grading	1.18 (1.04–1.33)	0.01
Tumor stage	1.31 (0.92–1.85)	n.s.
Nodal metastases	1.19 (1.18–2.72)	0.007
Sarcopenia***	1.55 (1.1–2.3)	0.023
Tumor grading	1.18 (1.04–1.33)	0.010
Tumor stage	1.31 (0.92–1.85)	n.s.
Nodal metastases	1.79 (1.18–2.72)	0.007
BMI	1.02 (0.98–1.06)	n.s.
Sarcopenia****	1.49 (1.01–2.19)	0.045
Tumor grading	1.17 (1.04–1.33)	0.011
Tumor stage	1.30 (0.91–1.84)	n.s.
Nodal metastases	1.75 (1.16–2.66)	0.008
Sex	1.04 (0.72–1.52)	n.s.
Interaction models**		***p*-value**
Sarcopenia*BMI	n.a.	0.018
Sarcopenia*sex	n.a.	0.032

*Charlson Age-Comorbidity Index

**adjusted for tumor grading, stage and nodal metastases

***adjusted for tumor grading/stage and nodal metastases as well as BMI

****adjusted for tumor grading/stage and nodal metastases as well as sex; n.s. denotes not significant, n.a. denotes not applicable.

Recurrence-free survival (RFS) was not impaired in sarcopenic vs. non-sarcopenic patients (Kaplan Meier/log rank: 9 vs. 10 months, *p =* 0.275, **S1 Fig**). Furthermore, no differences were found between male and female sarcopenic vs. non-sarcopenic patients (Kaplan Meier/log rank: male: 9 vs. 9 months, *p =* 0.216, and female: 10 vs. 11 months, *p =* 0.709; **S2 and S3 Figs**) or sarcopenic obese vs. non-sarcopenic obese as well as sarcopenic normal/underweight vs. non-sarcopenic normal/underweight patients (Kaplan Meier/log rank: BMI ≥25kg/m^2^: 9 vs. 13 months, *p =* 0.173 and BMI *<*25kg/m^2^: 9 vs. 10 months, *p = 0*.698, respectively, **S4 and S5 Figs)**.

## Discussion

Surgery is considered to be the mainstay of curative treatment for patients with PDAC. However, despite multimodal treatment strategies survival remains poor[1]. Yet, approved markers for predicting survival in PDAC are limited to tumor-specific features without considering host-specific body composition as significant determinant of outcome[2, 36–38]. In the present study, we aimed to validate sarcopenia and sarcopenic obesity as prognostic factors in a series of 133 patients undergoing surgery for PDAC in a national pancreatic centre. Our findings confirmed a negative impact of sarcopenia as well as sarcopenic obesity on overall survival (OS).

PDAC is associated with an excessive catabolic state that leads to progressive physical deterioration, known as cachexia, that reduces the tolerance to preoperative treatment and increases the likelihood of complications[39, 40]. PDAC patients are diagnosed with cachexia at a high rate and up to 80% suffered from severe cachexia at the time of death[41–43]. Cachexia is defined as a significant degree of weight loss depending on the prevalence of sarcopenia (generalized muscle disorder) and/or individual BMI[12] and is currently understudied in patients with PDAC[44]. In this study we demonstrate that preoperative sarcopenia and weight loss were significantly associated, whereby 62.8% of the sarcopenic patients suffered from weight loss >5% prior to surgery and hence suffered from cachexia[12].

Initially known as depletion of skeletal muscle mass, strength and physical performance, sarcopenia has recently been re-defined as a progressive and generalized skeletal muscle disorder associated with an increased likelihood of adverse outcomes and mortality[45] that results in high personal, social and economic costs[46]. Recent studies using computertomography for body composition analysis, validate that PDAC patients frequently suffer from sarcopenia[27, 44] and whenever possible, tumor resection is the best way to stop further muscle wasting[47]. In this study, more than half the patients (58.6%) with resectable pancreatic cancer were sarcopenic at the time of diagnosis, with male patients more likely to be affected (65.4%). Interestingly, we found a strong association of sarcopenia with neoadjuvant therapy, poor tumor differentiation and low serum albumin levels; all of these factors are known to further promote physical deterioration[31, 47].

Over the course of this past decade the clinical importance of sarcopenia as part of the cancer cachexia syndrome has been widely recognized and efforts have been made to define therapeutic improvements[48–54]. While originally a measure of frailty in geriatric non-cancer patients, the prognostic impact of sarcopenia has recently been observed in several gastrointestinal malignancies including PDAC[4, 5, 27]. The prevalence of sarcopenia found in these previous studies was consistent with our findings[27, 44].

We further validate that sarcopenia is an independent prognostic factor of OS and that its prognostic significance is associated with BMI. Patients suffering from sarcopenia who had a BMI ≥25 kg/m^2^ died sooner than normal or underweight patients. Peng et al. showed that sarcopenic patients undergoing pancreatic resection suffered from impaired OS[55]; still, no conclusion can be drawn as to the role of obesity in this study, since body composition data was not compared between BMI groups. Only a relatively small subset of studies report sarcopenia in the context of overweight patients and outline that the development of sarcopenic obesity and its modification options are barely understood[13, 34, 56]. Sarcopenic obesity has been found to be connected to aging and lifestyle. It is understood to be a complex syndrome that is based on reduced physical activity and results in accelerated loss of muscle mass and in decreased energy consumption. In cancer patients, increased body fat mass was associated with dissatisfactory pain management[13, 57]. In patients with resectable PDAC, sarcopenia, as well as sarcopenic obesity have been confirmed as crucial determinants of overall survival[56]; yet, compared to other gastrointestinal cancers, convincing data on the impact of body composition on postoperative complications are rare[58–60]. Here, we demonstrate that sarcopenic patients suffering from obesity (BMI ≥25kg/m^2^) have a higher rate of treatment-relevant postoperative complications. Similar findings have recently been reported by Sandini et al. revealing that sarcopenic obesity (referred to as visceral fat to skeletal muscle ratio) was an independent determinant of major complications after pancreaticoduodenectomy[24]. In the past, sarcopenic obesity has often been referred to as visceral fat to skeletal muscle ratio. Pecorelli et al. showed that the ratio of visceral fat to skeletal muscle (as an equivalent to sarcopenic obesity) was higher in patients who died after pancreatic resection compared to survivors[22]. However, the relationship between fat and muscle compartments seems to have an impact on outcome of PDAC patients and is currently understudied[18, 19, 25, 61]. Patients with sarcopenic obesity are less likely to be recognized as “high risk” patients, and hence less likely to receive (perioperative) nutritional support. Whether these patients should be supplied with tailored nutritional support in accordance with normal or underweight sarcopenic patients remains to be clarified by prospective clinical investigations. In fact, 64% of sarcopenic obese patients in this study indicated weight loss at the time of hospital admission, still weight loss was not significantly associated with sarcopenic obesity. Ultimately, these findings underline the importance of carefully screening this subgroup of patients.

According to previous findings, we found a strong association of sex with sarcopenia as well as sarcopenic obesity in patients with resectable PDAC[62]. In our patient cohort, males suffer from sarcopenia and sarcopenic obesity at a significantly higher rate. In fact, men experience greater muscle loss during the process of physiological aging[63]. We here demonstrate that the prognostic significance of sarcopenia is associated with sex. Male sarcopenic patients died sooner than male non-sarcopenic patients; in females, no differences were found between sarcopenic and non-sarcopenic patients. Studies in cancer patients show that men lose skeletal muscle mass and strength over time and at a faster rate when compared to women[64] implicating sex differences in body composition. Accordingly, sex-specific cut-offs have been recommended for body composition analysis[11].

The negative sequelae of sarcopenia have shown an increased awareness in regard to cancer treatment and studies exploring nutritional and pharmacological support as well as exercise programs to prevent physical deterioration are under investigation [7, 10, 65, 66]. Further results need to be looked at in order to define patient cohorts amenable for such multimodal approaches.

A considerable limitation of the study design is that since it was not based on a power calculation, subgroup analyses in particular have to be interpreted deliberately.

Our findings confirm that sarcopenia and sarcopenic obesity are independent prognostic factors for OS in patients with resectable PDAC. In sarcopenic patients, neoadjuvant therapy and tumor grade as well as sex, nutritional status, weight loss and BMI play a crucial role for postoperative outcome. These data underline the importance of body composition in addition to other determinants of the disease in differentiating a “high risk” situation from a conditional perspective and selecting specifically those patients that might benefit from additional support strategies.

## Supporting information

S1 Fig Kaplan Meier curve for recurrence-free survival in sarcopenic vs. non-sarcopenic patients. Sarcopenia does not impair recurrence-free survival in patients with resectable PDAC (15 vs. 25 months, *p =* 0.275).
(TIFF)

S2 Fig Kaplan Meier curve for recurrence-free survival in male sarcopenic vs. non-sarcopenic patients. Sarcopenia does not impair recurrence-free survival in male patients with resectable PDAC (9 vs. 9 months, *p =* 0.216).
(TIFF)

S3 Fig Kaplan Meier curve for recurrence-free survival in female sarcopenic vs. non sarcopenic patients. Sarcopenia does not impair recurrence-free survival in female patients with resectable PDAC (10 vs. 11 months, *p =* 0.709).
(TIFF)

S4 Fig Kaplan Meier curve for recurrence-free survival in obese sarcopenic vs. non sarcopenic patients (BMI ≥25 kg/m^2^). Sarcopenia does not impair recurrence-free survival in obese patients with resectable PDAC (9 vs. 10 months, *p =* 0.698).
(TIFF)

S5 Fig Kaplan Meier curve for recurrence-free survival in normal/underweight sarcopenic vs. non-sarcopenic patients (BMI *<*25 kg/m^2^). Sarcopenia does not impair recurrence-free survival in normal/underweight patients with resectable PDAC (9 vs. 13 months, *p =* 0.173).
(TIFF)
