# Correction: Sarcopenia and sarcopenic obesity are independent adverse prognostic factors in resectable pancreatic ductal adenocarcinoma

**DOI:** 10.1371/journal.pone.0244896

**Published:** 2020-12-29

**Authors:** Elisabeth S. Gruber, Gerd Jomrich, Dietmar Tamandl, Michael Gnant, Martin Schindl, Klaus Sahora

There are errors in the author affiliations. The correct affiliations are as follows:

Elisabeth S. Gruber^1.3^, Gerd Jomrich^1,3^, Dietmar Tamandl^2^, Michael Gnant^3^, Martin Schindl^1,3^, Klaus Sahora^1,3^

**1** Division of General Surgery, Department of Surgery, Medical University of Vienna, Vienna, Austria, **2** Department of Biomedical Imaging and Image-guided Therapy, Medical University of Vienna, Vienna, Austria, **3** Comprehensive Cancer Center, Medical University of Vienna, Vienna, Austria.
