# Supplementary figures and images for: Sarcopenia and sarcopenic obesity are independent adverse prognostic factors in resectable pancreatic ductal adenocarcinoma
Source: PLoS One. 2019 May 6;14(5):e0215915. doi: 10.1371/journal.pone.0215915 (PMC6502449; doi:10.1371/journal.pone.0215915)

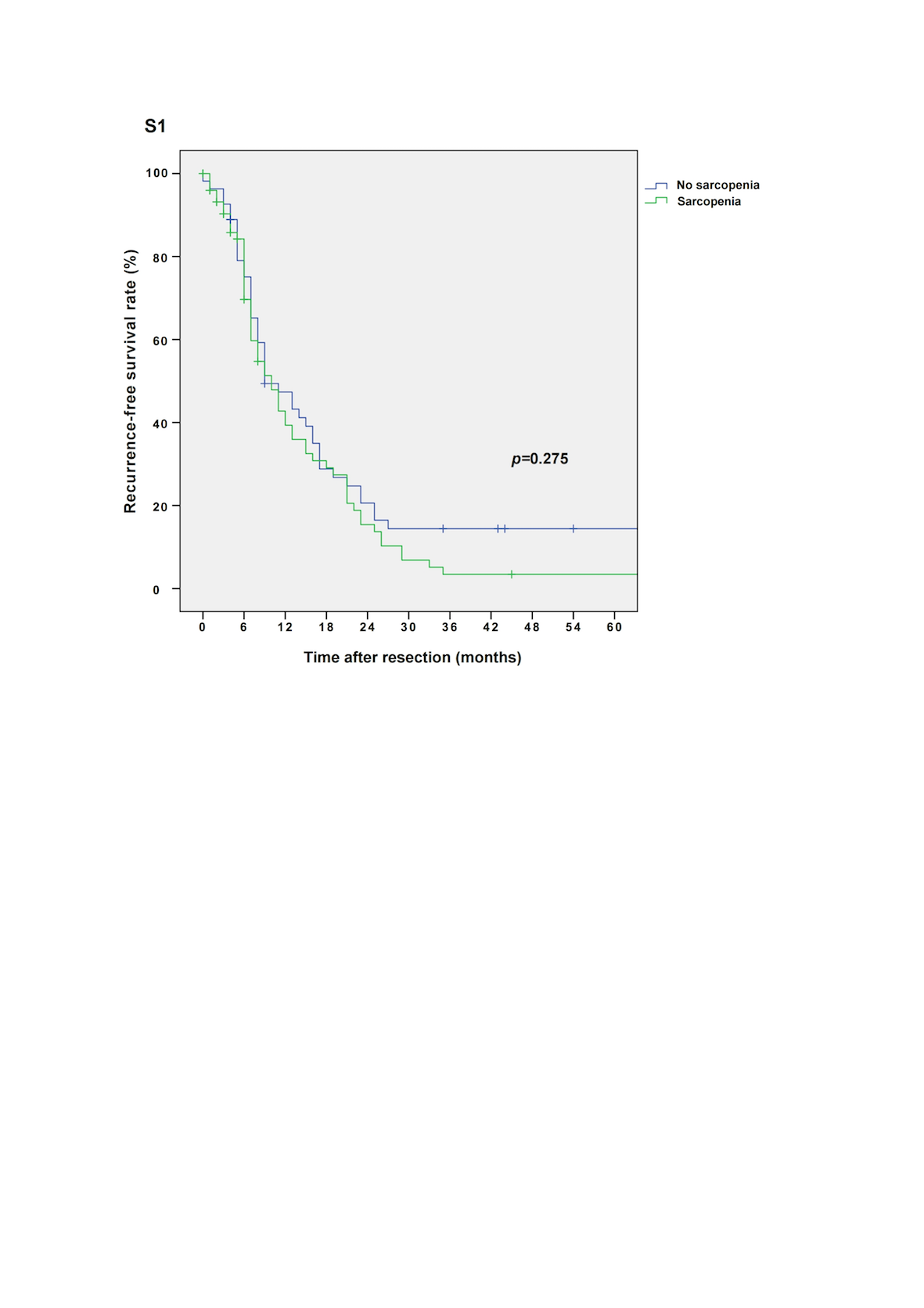

Supplement: S1 Fig — Sarcopenia does not impair recurrence-free survival in patients with resectable PDAC (15 vs. 25 months, p = 0.275). (TIFF) [file pone.0215915.s001.tiff]

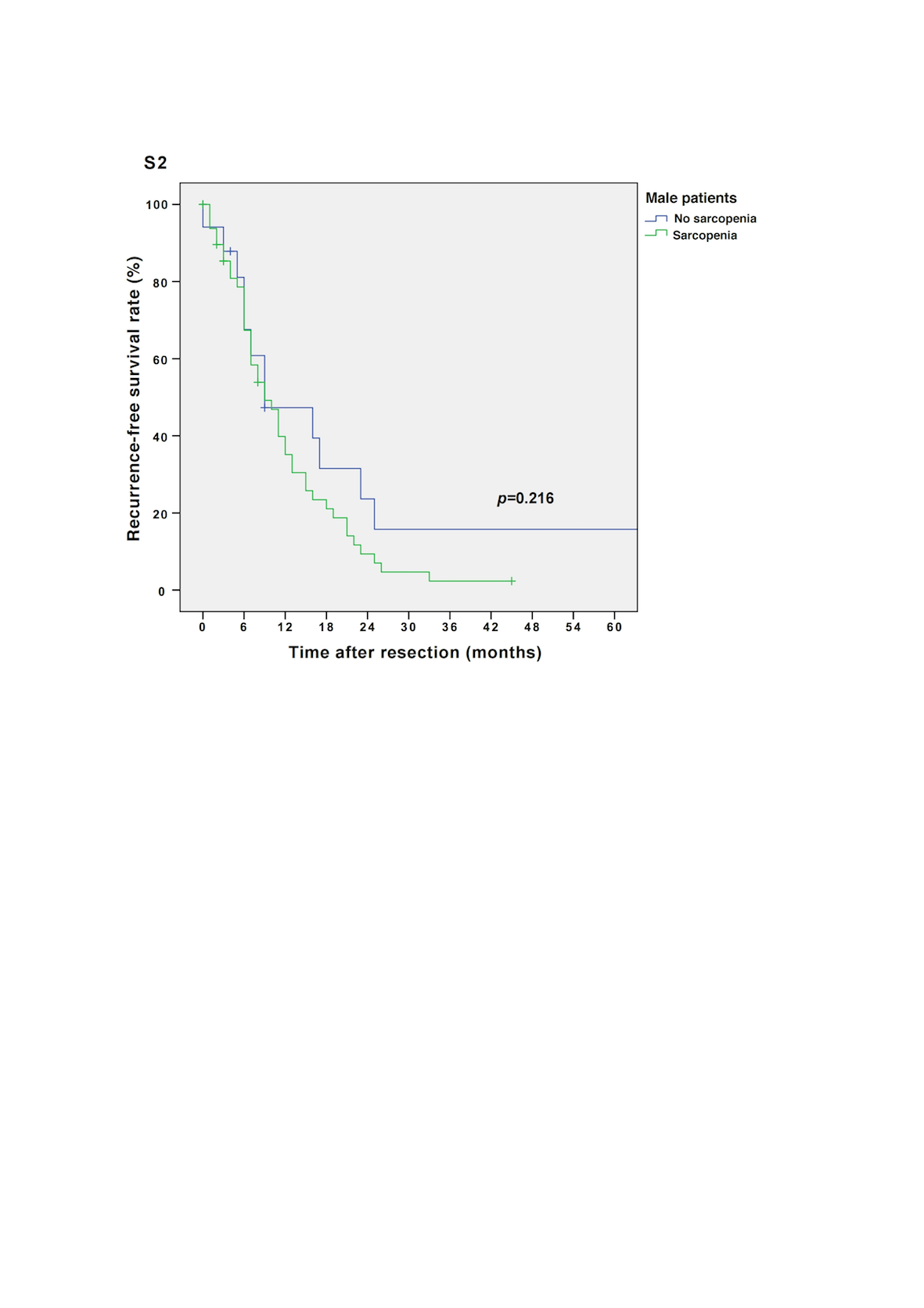

Supplement: S2 Fig — Sarcopenia does not impair recurrence-free survival in male patients with resectable PDAC (9 vs. 9 months, p = 0.216). (TIFF) [file pone.0215915.s002.tiff]

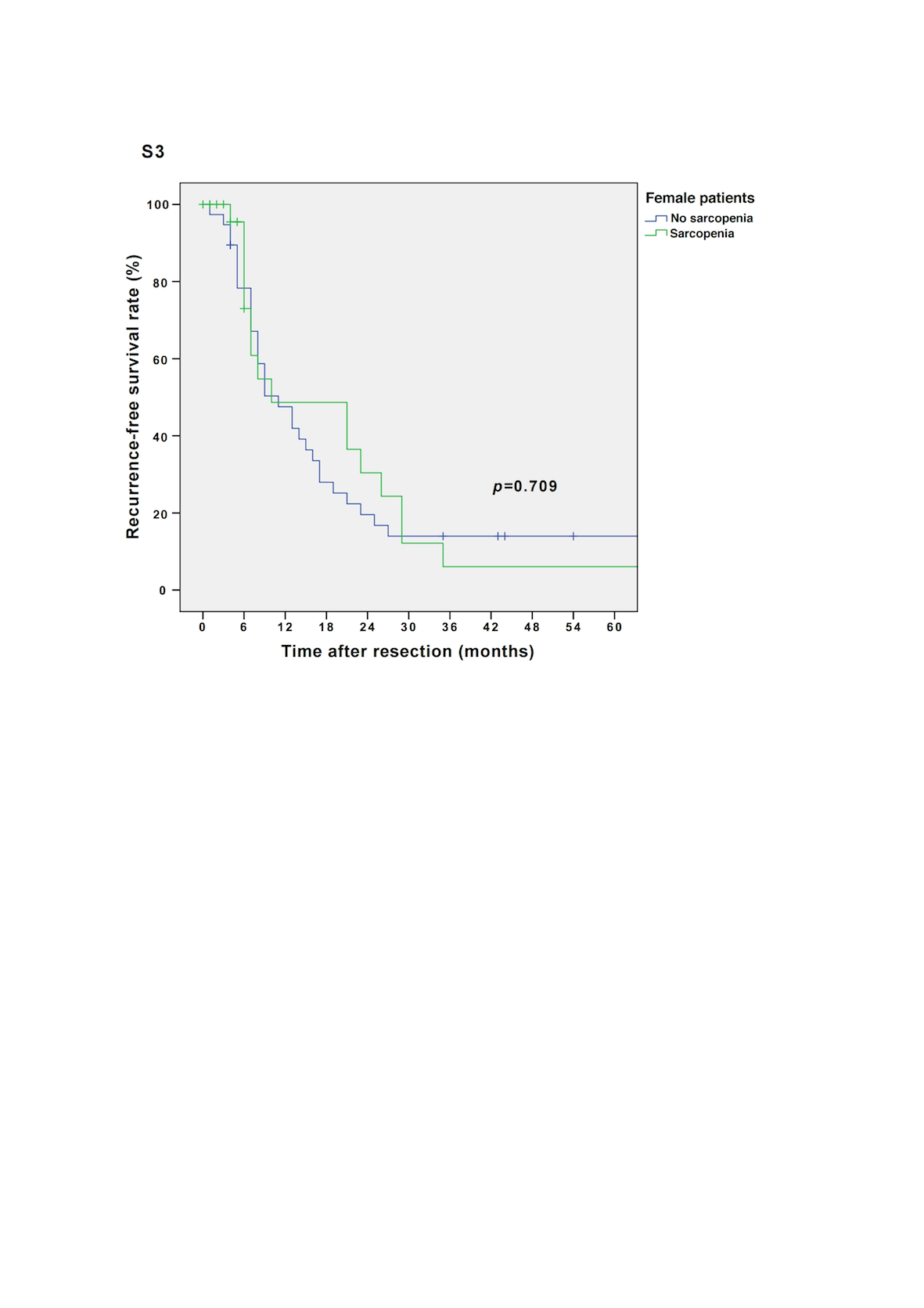

Supplement: S3 Fig — Sarcopenia does not impair recurrence-free survival in female patients with resectable PDAC (10 vs. 11 months, p = 0.709). (TIFF) [file pone.0215915.s003.tiff]

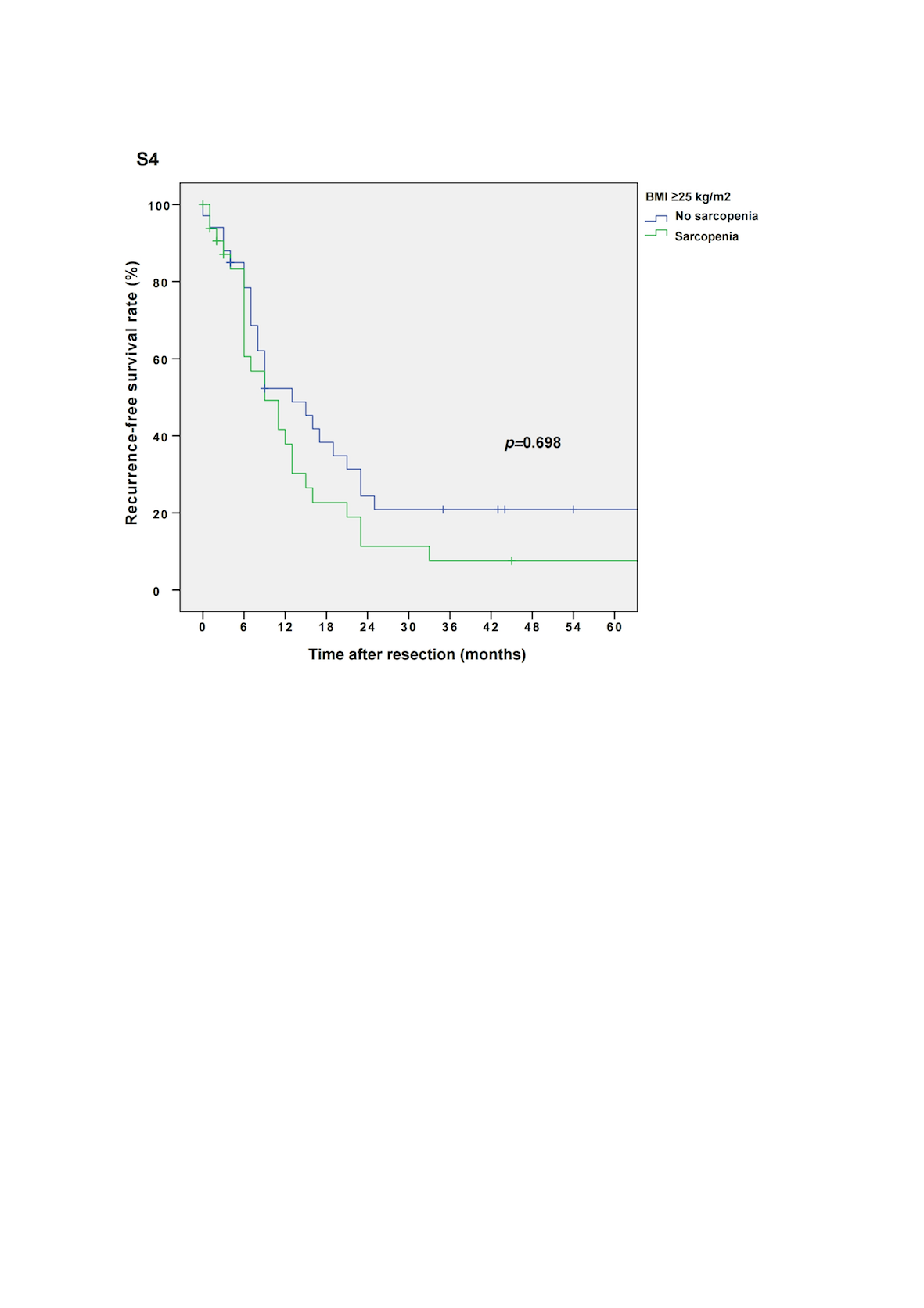

Supplement: S4 Fig — Sarcopenia does not impair recurrence-free survival in obese patients with resectable PDAC (9 vs. 10 months, p = 0.698). (TIFF) [file pone.0215915.s004.tiff]

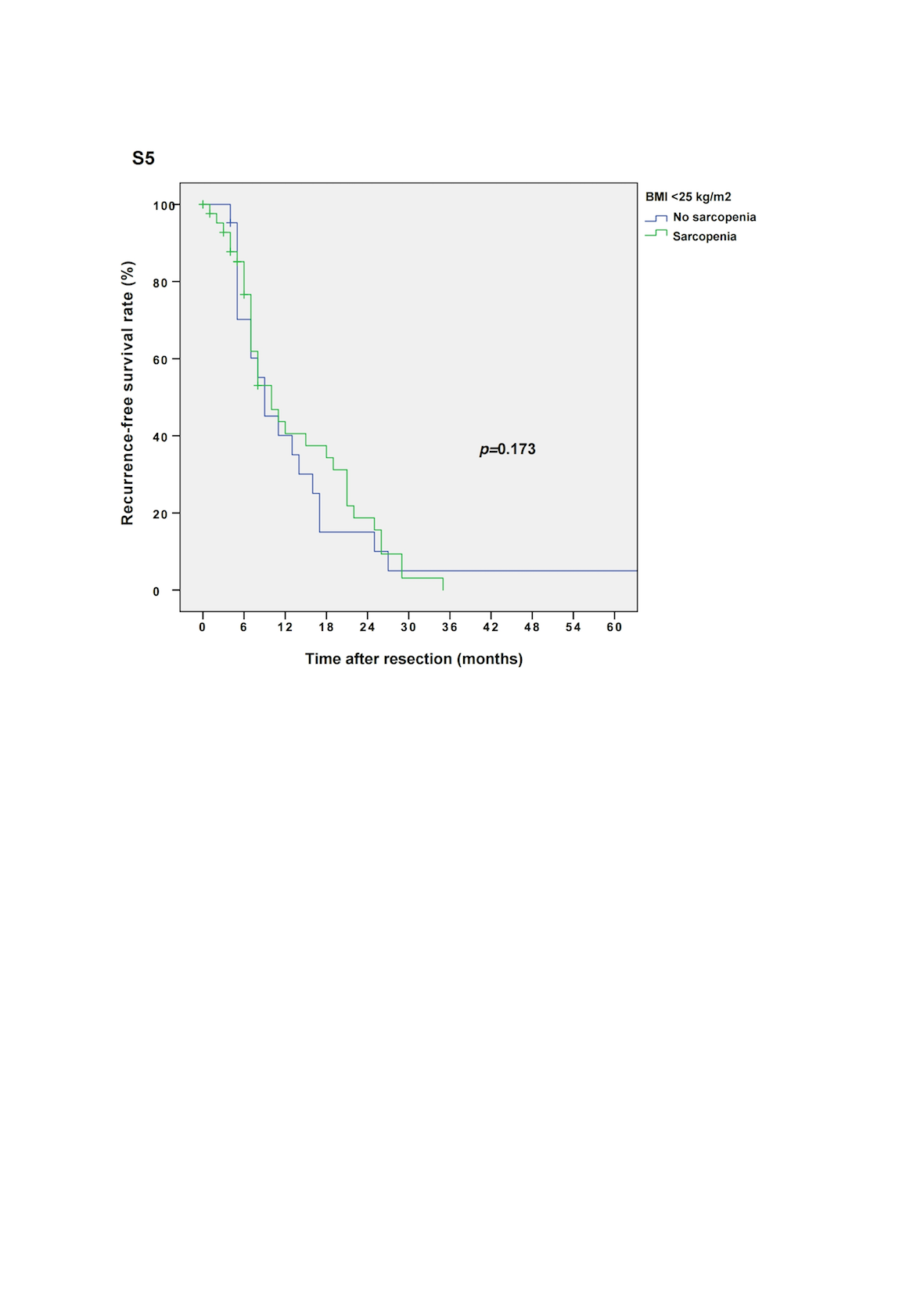

Supplement: S5 Fig — Sarcopenia does not impair recurrence-free survival in normal/underweight patients with resectable PDAC (9 vs. 13 months, p = 0.173). (TIFF) [file pone.0215915.s005.tiff]
